# Association between Life’s Crucial 9 and overactive bladder: the mediating role of weight-adjusted-waist index

**DOI:** 10.3389/fnut.2024.1508062

**Published:** 2025-01-06

**Authors:** Hongyang Gong, Shuqin Duan, Shaoqun Huang

**Affiliations:** ^1^Department of Oncology Surgery, Fuzhou Hospital of Traditional Chinese Medicine Affiliated to Fujian University of Traditional Chinese Medicine, Fuzhou, Fujian Province, China; ^2^Department of Physiology, College of Medicine, Chosun University, Gwangju, Republic of Korea; ^3^Department of Obstetrics and Gynaecology, The Second Hospital of Jilin University, Changchun, Jilin Province, China

**Keywords:** Life’s Crucial 9, overactive bladder, weight-adjusted-waist index, NHANES, mediation analysis

## Abstract

**Background:**

Research suggests a potential connection between cardiovascular health, obesity, and overactive bladder (OAB). However, the mechanisms by which obesity influences the relationship between cardiovascular health and OAB remain unclear. Life’s Crucial 9 (LC9) is a recently proposed method for assessing cardiovascular health, while the weight-adjusted waist index (WWI) is a novel and more accurate measure of obesity. This study investigates the relationship between LC9 and OAB and assesses whether WWI moderates this relationship.

**Methods:**

Data for this study came from the National Health and Nutrition Examination Survey (NHANES). We used subgroup analyses, restricted cubic spline curves (RCS), and multivariate logistic regression to explore the relationship between LC9 and OAB. Additionally, mediation analysis was conducted to investigate the potential association between WWI levels and the relationship between LC9 and OAB.

**Results:**

A total of 25,319 participants were included in this study, among which 5,038 reported incidents of OAB. After adjusting for all variables using multivariable logistic regression, an increase of 10 units in LC9 was associated with a 28% reduction in the incidence of OAB (OR = 0.72, 95% CI: 0.69, 0.76), while an increase of one unit in WWI was associated with a 40% increase in the incidence of OAB (OR = 1.40, 95% CI: 1.29, 1.51). Consistent results were also observed when LC9 and WWI were categorized into quartiles, with a P for trend <0.001. The analysis using restricted cubic splines indicated a linear negative correlation between the incidence of OAB and LC9. Mediation analysis revealed that 13.89% of the relationship between LC9 and OAB was mediated by WWI (*p* = 0.002).

**Conclusion:**

This study found a significant negative correlation between LC9 and OAB, with WWI acting as a partial mediator in this relationship. This study provides new insights for future research into the relationship between LC9 and OAB and the role of WWI as a mediator.

## Introduction

Overactive bladder (OAB) is defined as a storage symptom syndrome characterized by urgency, with or without urgency urinary incontinence (UUI), typically accompanied by daytime frequency and nocturia (1). OAB is prevalent in both men and women, with 12.8% of women and 10.8% of men reporting lower urinary tract symptoms (LUTS) that define OAB (2, 3). It is a chronic debilitating condition that reportedly affects the quality of life of over 30 million Americans. In the United States, underdiagnosis and inadequate treatment of OAB contribute to increased healthcare costs, with estimates indicating that direct and indirect medical costs could reach $86 billion annually by 2020 (4). The etiology of OAB is complex, and its pathophysiology remains unclear; however, chronic systemic inflammation and bladder urothelial inflammation, including certain inflammatory proteins and cytokines, may trigger the onset of OAB (5). Several theories regarding the pathophysiology of OAB exist, including the myogenic hypothesis, urothelial dysfunction hypothesis, neurogenic hypothesis, and detrusor underactivity; nevertheless, a comprehensive understanding of the underlying mechanisms is still lacking (1).

Recent data indicate a global rise in overweight and obesity, with approximately 30% of the world’s population classified as overweight (6). In North America, one-third of adults are considered obese, and studies have shown that abdominal obesity is a strong predictor of metabolic disorders and cardiovascular disease (CVD) (7). While body mass index (BMI) is the most widely used measure of obesity, it primarily focuses on overall obesity rather than abdominal fat. To address this gap, the weight-adjusted waist index (WWI) was introduced in 2018. WWI is a unique obesity index that can predict the risks of cardiometabolic diseases, cardiovascular events, and all-cause mortality, demonstrating superior predictive ability (8, 9). Research has shown that WWI positively correlates with total fat area (TFA), subcutaneous fat area, and visceral fat area. Given that visceral fat is considered more inflammatory compared to subcutaneous fat, the correlation between fat mass and the incidence of CVD is stronger (10, 11). Evidence suggests that higher visceral fat is a risk factor for OAB, and reducing visceral abdominal fat may help alleviate OAB symptoms (12, 13).

Metabolic syndrome (MetS) is defined by the World Health Organization (WHO) as a pathological condition characterized by abdominal obesity, insulin resistance, hypertension, and dyslipidemia (14). Research has shown a close association between MetS and cardiovascular disease, with each component of MetS acting as an independent risk factor for cardiovascular events (15). Several studies have indicated a strong relationship between OAB and Mets (16, 17). In 2010, the American Heart Association (AHA) introduced a framework for assessing cardiovascular health behaviors and factors, termed Life’s Simple 7 (LS7) (18), which has since been refined. Recently, the AHA updated this framework, expanding the assessment of cardiovascular health behaviors and factors to include mental health, culminating in the introduction of Life’s Essential 8 (LE8) in 2022. This was further developed into Life’s Crucial 9 (LC9), which encompasses sleep, smoking, physical activity, diet, BMI, non-HDL cholesterol, blood glucose, blood pressure, and mental health (19, 20). Given that research suggests weight loss in abdominal obesity may improve MetS (21), and that WWI and the associated LC9 indicators can be modified through lifestyle changes, there may be new avenues for managing and predicting OAB. Considering that excess abdominal fat may exacerbate OAB symptoms and that WWI and LC9 reflect both metabolic status and cardiovascular health, there is potential for a better understanding of the mechanisms underlying OAB.

Based on the above, this study hypothesizes that WWI mediates the relationship between the LC9 and OAB. Specifically, LC9, as a comprehensive assessment of cardiovascular health, may have protective effects against OAB by promoting healthier behaviors (e.g., dietary patterns, physical activity, weight management) and optimizing biomarkers (e.g., blood pressure, and blood glucose levels). However, obesity, as a key modifiable risk factor, may attenuate the potential benefits of LC9 on OAB. WWI, a novel measure of obesity, reflects both fat distribution and its impact on metabolic health. This study posits that WWI is not only independently associated with the risk of OAB but also serves as a mediator in the relationship between LC9 and OAB. Through its mediating role, WWI may partially explain how the health benefits of LC9 are influenced by the degree and distribution of obesity. By conducting mediation analysis, this study aims to validate this hypothesis, offering new insights into the potential pathways linking LC9, WWI, and OAB. These findings could provide valuable evidence for developing prevention and intervention strategies for OAB. Using NHANES data from 2005 to 2018, this study analyzes the relationship between LC9 and OAB while evaluating the role of WWI as a mediator, which may pave the way for new directions in diagnosing and managing OAB.

## Methods

### Study participants

This cross-sectional study utilized data from the National Health and Nutrition Examination Survey (NHANES), conducted by the National Center for Health Statistics (NCHS), which aims to collect demographic data regarding the health and nutritional status of U.S. citizens. All NHANES study protocols were approved by the NCHS Research Ethics Review Board, and written informed consent was obtained from all survey participants. Our secondary analysis adhered to the STROBE guidelines for cross-sectional studies (22) and did not require additional approval from an institutional review board. For detailed information regarding NHANES methodology and ethical considerations, please visit the CDC and NCHS websites.^1^

**Figure 1:**
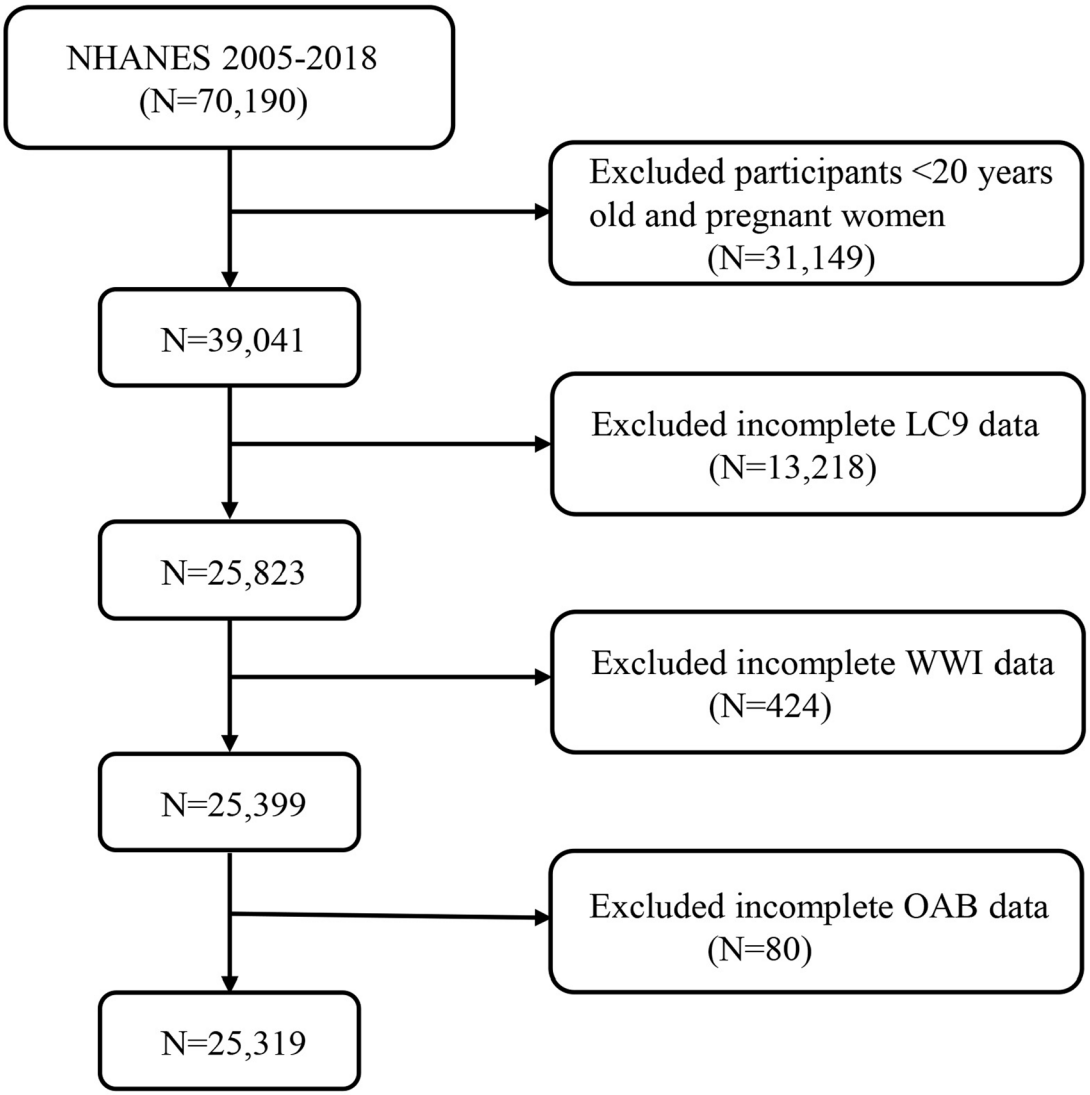
A flow diagram of eligible participant selection in the National Health and Nutrition Examination Survey. LC9, Life’s Crucial 9; OAB, overactive bladder; WWI, weight-adjusted-waist index.

In this cross-sectional study, nationally representative data from the National Health and Nutrition Examination Survey (NHANES) were utilized. Among 70,190 participants aged 20 years and older from the seven NHANES cycles conducted between 2005 and 2018, 39,041 participants were identified, excluding those who were pregnant and those with incomplete data for the LC9 indicators (*n* = 13,218), WWI (*n* = 424), and OAB (*n* = 80). Ultimately, a total of 25,319 participants were included in the study (Figure 1).

### OAB assessment

In patients, urgency urinary incontinence and nocturia must be considered as indicators of OAB based on their definitions. We assessed urinary incontinence and nocturia using three specific questions from the NHANES questionnaires KIQ044, KIQ450, and KIQ480 (23): (1) In the past year, have you experienced involuntary leakage of urine accompanied by urgency or a feeling of pressure, making it difficult to reach the bathroom in time? (2) How often does this occur? (3) In the past month, how many times did you typically wake up to urinate between going to bed and getting up in the morning?

Subsequently, the Overactive Bladder Symptom Score (OABSS) questionnaire was used to quantify OAB (24). Detailed scoring criteria can be found in Supplementary Table S4. Each participant’s OABSS score was calculated by summing the scores for urgency urinary incontinence and nocturia. In this survey, participants with a total score of 3 or higher were considered to have overactive bladder.

### Definition of weight-adjusted-waist index

We obtained data on participants’ weight-adjusted waist index (WWI) from the NHANES database. WWI is calculated based on waist circumference (WC) and weight to assess central obesity. Each participant’s WWI was determined using the following formula: WWI = WC (cm)/(weight (kg)^2^) (25). In this study, WWI was utilized as a mediating variable. WWI was selected as a measure of central obesity due to its ability to adjust for both weight and waist circumference, factors that are commonly linked to central fat distribution. Unlike traditional indices like Body Mass Index (BMI), which does not account for fat distribution, or Waist-to-Height Ratio (WHtR), which adjusts for height but not for overall weight, WWI provides a more specific reflection of central obesity by incorporating both waist circumference and weight in a balanced way. This makes WWI a potentially more precise measure of abdominal adiposity and its association with conditions like OAB.

### Definition of Life’s Crucial 9

LC9 encompasses nine indicators: four health behaviors (healthy diet, physical activity, smoking cessation, and healthy sleep) and five health factors (weight management, cholesterol control, blood glucose management, blood pressure management, and mental health). Detailed instructions for calculating each participant’s LC9 score using the NHANES database are provided in Supplementary Table S1. In summary, each of the nine LC9 indicators is scored on a scale from 0 to 100. The LC9 score is determined by the average of the scores from the nine individual indicators. The 2015 Healthy Eating Index (HEI-2015) was utilized to assess diet quality (26). The components and scoring criteria for the HEI-2015 are detailed in Supplementary Table S2. Sleep health, smoking status, physical activity, and mental health data were derived from standardized questionnaires, while BMI, blood pressure, blood glucose, and cholesterol levels were obtained from trained professionals using the NHANES database (see Footnote 1).

### Covariables

The covariates in this study included age, sex, race, marital status, education level, poverty-to-income ratio (PIR), smoking, alcohol consumption, hypertension, diabetes, and dyslipidemia. Detailed information regarding these covariates can be found in Supplementary Table S3.

### Statistical analysis

Statistical analyses were conducted using R software (version 4.3.1), with all analyses employing sampling weights to ensure that the estimated data were nationally representative. In our study, “WTMEC2YR” was used as the weighting variable, and the new weight (2005–2018) was calculated as 1/7 × WTMEC2YR (27). Continuous variables were expressed as mean ± standard deviation, and *p*-values were calculated using t-tests. The percentages and p-values for categorical variables (weighted N, %) were computed using weighted chi-square tests. Multivariable logistic regression models were used to analyze the relationships between LC9 and OAB, as well as WWI and OAB, constructing three models: (1) a crude model without covariate adjustment, (2) a model adjusted for age, sex, education level, marital status, poverty-to-income ratio (PIR), and race, and (3) a model further adjusted for smoking, alcohol consumption, hypertension, diabetes, and dyslipidemia. Smoothing spline fitting was employed to explore the linear or nonlinear relationship between LC9 and OAB. Subgroup analyses were performed to examine the risk stratification of the relationship between LC9 and OAB across various subgroups. Mediation analysis was conducted to evaluate the indirect, direct, and overall effects of WWI on the relationship between LC9 and OAB. The mediation proportion was calculated as (indirect effect / (indirect effect + direct effect)) × 100%. Mediation effects were estimated using the “mediation” package in R software (27). A two-sided *p*-value of less than 0.05 was considered statistically significant.

## Results

### Baseline characteristics

This study included 25,319 participants aged 20 and older, representing approximately 159.5 million adults in the United States. The prevalence of OAB was found to be 16%, affecting around 24.87 million individuals. Statistically significant differences (*p* < 0.05) were observed between OAB patients and non-OAB individuals concerning age, sex, race, marital status, education level, income level, smoking, alcohol consumption, hypertension, diabetes, and dyslipidemia. Additionally, the LC9 levels in the OAB group were lower than those in the non-OAB group, while the WWI was higher in the OAB group compared to the non-OAB group. Further details can be found in Table 1.

**Table 1:** Baseline characteristics of all participants were stratified by OAB, weighted.

Characteristic	Overall, *N* = 159,501,902 (100%)	Non-OAB, *N* = 134,630,077 (84%)	OAB, *N* = 24,871,825 (16%)	*p* value
No. of participants in the sample	25,319	20,281	5,038	**–**
Age (%)				**<0.001**
20–40	57,778,401 (36%)	54,076,678 (40%)	3,701,723 (15%)	
41–60	62,050,346 (39%)	52,680,119 (39%)	9,370,227 (38%)	
>60	39,673,155 (25%)	27,873,280 (21%)	11,799,876 (47%)	
Gender (%)				**<0.001**
Male	77,790,801 (49%)	68,407,709 (51%)	9,383,092 (38%)	
Female	81,711,101 (51%)	66,222,368 (49%)	15,488,733 (62%)	
Race (%)				**<0.001**
Non-Hispanic White	112,486,043 (71%)	95,677,264 (71%)	16,808,779 (68%)	
Non-Hispanic Black	15,970,245 (10%)	12,099,397 (9.0%)	3,870,848 (16%)	
Other	18,688,523 (12%)	16,153,948 (12%)	2,534,575 (10%)	
Mexican American	12,357,091 (7.7%)	10,699,469 (7.9%)	1,657,622 (6.7%)	
Married/live with partner (%)				**<0.001**
No	55,571,209 (35%)	45,697,640 (34%)	9,873,569 (40%)	
Yes	103,930,693 (65%)	88,932,437 (66%)	14,998,256 (60%)	
Education level (%)				**<0.001**
Below high school	22,365,942 (14%)	16,758,426 (12%)	5,607,515 (23%)	
High School or above	137,135,961 (86%)	117,871,651 (88%)	19,264,310 (77%)	
PIR (%)				**<0.001**
Not Poor	121,352,444 (81%)	104,362,218 (82%)	16,990,225 (74%)	
Poor	28,432,294 (19%)	22,449,409 (18%)	5,982,885 (26%)	
Unknown	9,717,165	7,818,450	1,898,715	
Smoking (%)				**<0.001**
Never	87,672,025 (55%)	75,694,251 (56%)	11,977,774 (48%)	
Former	41,201,305 (26%)	33,449,858 (25%)	7,751,447 (31%)	
Current	30,628,573 (19%)	25,485,968 (19%)	5,142,605 (21%)	
Drinking (%)				**<0.001**
Former	20,762,603 (13%)	15,772,884 (12%)	4,989,719 (21%)	
Heavy	32,083,850 (21%)	28,591,397 (22%)	3,492,453 (15%)	
Mild	58,836,357 (38%)	50,240,302 (38%)	8,596,055 (36%)	
Moderate	27,867,147 (18%)	24,427,341 (19%)	3,439,807 (14%)	
Never	15,854,359 (10%)	12,622,386 (9.6%)	3,231,973 (14%)	
Hypertension (%)				**<0.001**
No	99,348,284 (62%)	89,218,267 (66%)	10,130,017 (41%)	
Yes	60,153,618 (38%)	45,411,810 (34%)	14,741,808 (59%)	
Diabetes (%)				**<0.001**
No	139,860,299 (88%)	121,187,653 (90%)	18,672,646 (75%)	
Yes	19,641,603 (12%)	13,442,424 (10.0%)	6,199,179 (25%)	
High cholesterol (%)				**<0.001**
No	88,242,935 (62%)	76,819,268 (65%)	11,423,666 (49%)	
Yes	54,062,223 (38%)	42,234,405 (35%)	11,827,818 (51%)	
Mean LC9 score [mean (SD)]	70.75 (13.48)	72.07 (12.94)	63.56 (14.07)	**<0.001**
Mean HEI-2015 diet score [mean (SD)]	39.40 (31.31)	39.39 (31.35)	39.43 (31.08)	0.876
Mean physical activity score [mean (SD)]	72.33 (40.76)	74.38 (39.55)	61.21 (45.16)	**<0.001**
Mean tobacco exposure score [mean (SD)]	71.36 (38.70)	71.88 (38.65)	68.56 (38.81)	**<0.001**
Mean sleep health score [mean (SD)]	83.55 (24.11)	84.41 (23.32)	78.91 (27.56)	**<0.001**
Mean psychological health score [mean (SD)]	89.51 (22.99)	91.42 (20.74)	79.21 (30.61)	**<0.001**
Mean body mass index score [mean (SD)]	60.67 (33.46)	62.48 (32.94)	50.87 (34.52)	**<0.001**
Mean blood lipid score [mean (SD)]	64.35 (30.27)	64.90 (30.32)	61.36 (29.82)	**<0.001**
Mean blood glucose score [mean (SD)]	86.07 (23.95)	88.05 (22.32)	75.34 (29.08)	**<0.001**
Mean blood pressure score [mean (SD)]	69.47 (30.84)	71.75 (29.96)	57.12 (32.55)	**<0.001**
LC9 (%)				**<0.001**
T1	54,510,078 (34%)	40,735,774 (30%)	13,774,304 (55%)	
T2	50,005,789 (31%)	43,417,118 (32%)	6,588,671 (26%)	
T3	54,986,035 (34%)	50,477,185 (37%)	4,508,851 (18%)	
WWI [mean (SD)]	10.96 (0.82)	10.88 (0.80)	11.39 (0.81)	**<0.001**
WWI (%)				**<0.001**
T1	53,166,733 (33%)	49,287,521 (37%)	3,879,212 (16%)	
T2	53,172,285 (33%)	45,610,157 (34%)	7,562,129 (30%)	
T3	53,162,884 (33%)	39,732,399 (30%)	13,430,485 (54%)	

Mean (SD) for continuous variables: the *p* value was calculated by the weighted student’s *t*-test. LC9, Life’s Crucial 9; OAB, overactive bladder; WWI, weight-adjusted-waist index; HEI-2015, Healthy Eating Index-2015; PIR, Ratio of family income to poverty. Bold values indicate *p* < 0.05.

### Association between LC9, WWI, and OAB

**Figure 2:**
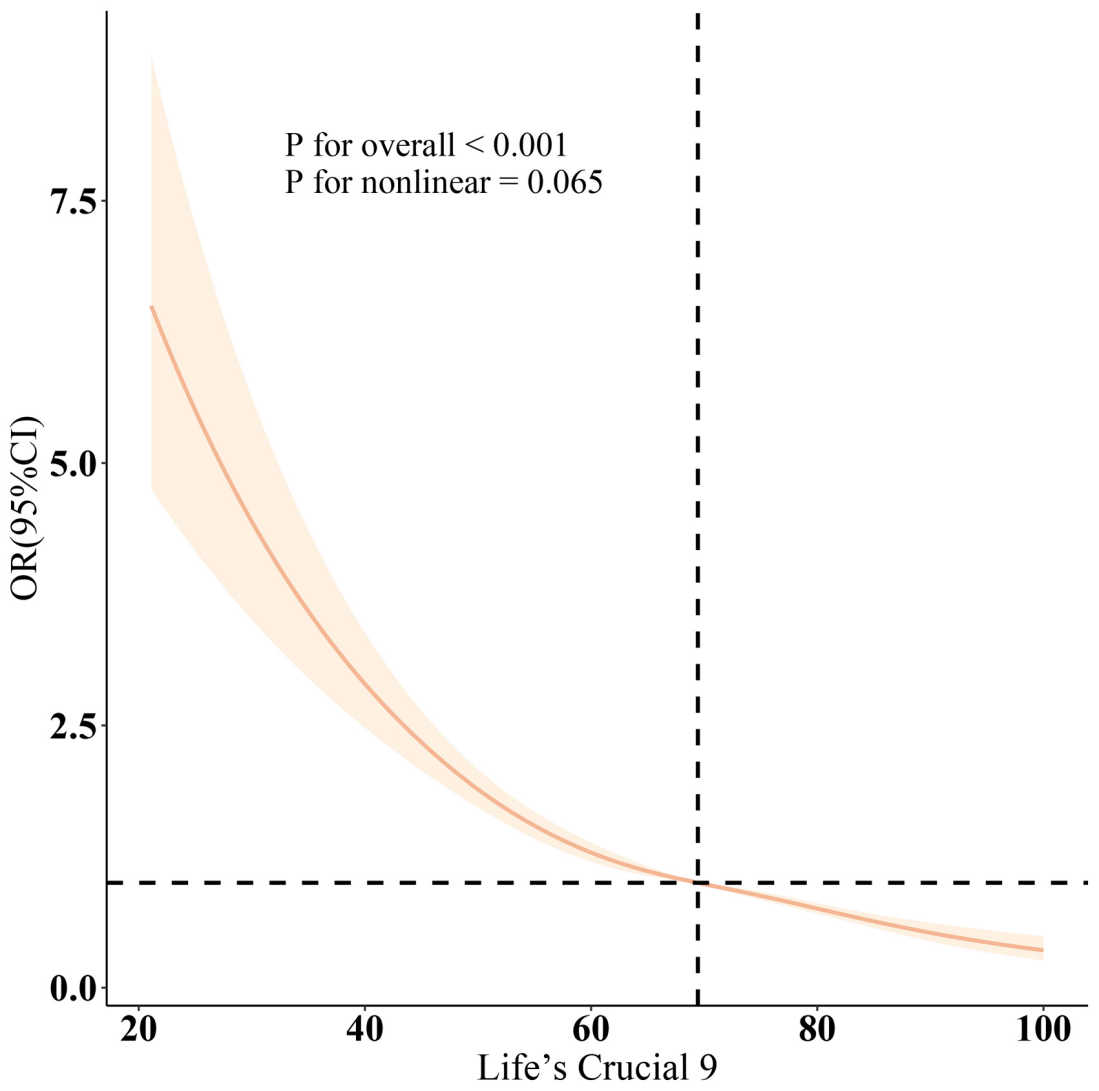
Dose–response relationships between LC9 and OAB. OR (solid lines) and 95% confidence levels (shaded areas) were adjusted for age, gender, education level, marital, PIR, race, smoking, drinking, hypertension, diabetes, and high cholesterol.

As shown in Table 2, three different models were employed to evaluate the association between LC9 and the prevalence of OAB, all indicating a negative correlation between LC9 and OAB prevalence (all *p* < 0.001). In Model 3, after adjusting for various covariates, each 10-point increase in LC9 was associated with a 28% reduction in OAB prevalence [odds ratio: 0.72 (95% confidence interval: 0.69, 0.76)]. Furthermore, when categorized into tertiles, the group with the highest LC9 scores (T3) exhibited a 53% lower prevalence of OAB compared to the group with the lowest scores (T1) [odds ratio: 0.47 (95% confidence interval: 0.40, 0.54)]. Additionally, the relationship between WWI and OAB was assessed, revealing a positive correlation across all three models (all *p* < 0.001). Higher WWI scores were associated with increased OAB prevalence, with statistically significant results (all *p* < 0.05). The results from the restricted cubic spline (RCS) analysis (Figure 2) further illustrated a significant linear negative correlation between LC9 and OAB prevalence after adjusting for relevant variables (overall *p* < 0.001; nonlinear *p* = 0.065).

**Table 2:** Association between LC9, WWI, and OAB, NHANES 2005–2018.

Characteristics	Model 1 [OR (95% CI)]	*p-*value	Model 2 [OR (95% CI)]	*p-*value	Model 3 [OR (95% CI)]	*p-*value
LC9-OAB						
Continuous (per 10 scores)	0.63 (0.61, 0.65)	<0.001	0.70 (0.67, 0.73)	<0.001	0.72 (0.69, 0.76)	<0.001
Tertile						
T1	1 (ref.)		1 (ref.)		1 (ref.)	
T2	0.45 (0.40, 0.50)	<0.001	0.54 (0.48, 0.60)	<0.001	0.61 (0.53, 0.69)	<0.001
T3	0.26 (0.23, 0.30)	<0.001	0.38 (0.33, 0.43)	<0.001	0.47 (0.40, 0.54)	<0.001
*P* for trend	<0.001		<0.001		<0.001	
WWI-OAB						
Continuous	2.18 (2.06, 2.31)	<0.001	1.59 (1.48, 1.71)	<0.001	1.40 (1.29, 1.51)	<0.001
Tertile						
T1	1 (ref.)		1 (ref.)		1 (ref.)	
T2	2.11 (1.86, 2.39)	<0.001	1.58 (1.38, 1.80)	<0.001	1.54 (1.31, 1.79)	<0.001
T3	4.30 (3.80, 4.85)	<0.001	2.31 (1.99, 2.69)	<0.001	1.90 (1.59, 2.28)	<0.001
*P* for trend	<0.001		<0.001		<0.001	

Model 1: no covariates were adjusted. Model 2: age, gender, education level, marital, PIR, and race were adjusted. Model 3: age, gender, education level, marital, PIR, race, smoking, drinking, hypertension, diabetes, and high cholesterol were adjusted. LC9, Life’s Crucial 9; OAB, overactive bladder; WWI, weight-adjusted-waist index; PIR, Ratio of family income to poverty; OR, odds ratio; CI, confidence interval.

**Figure 3:**
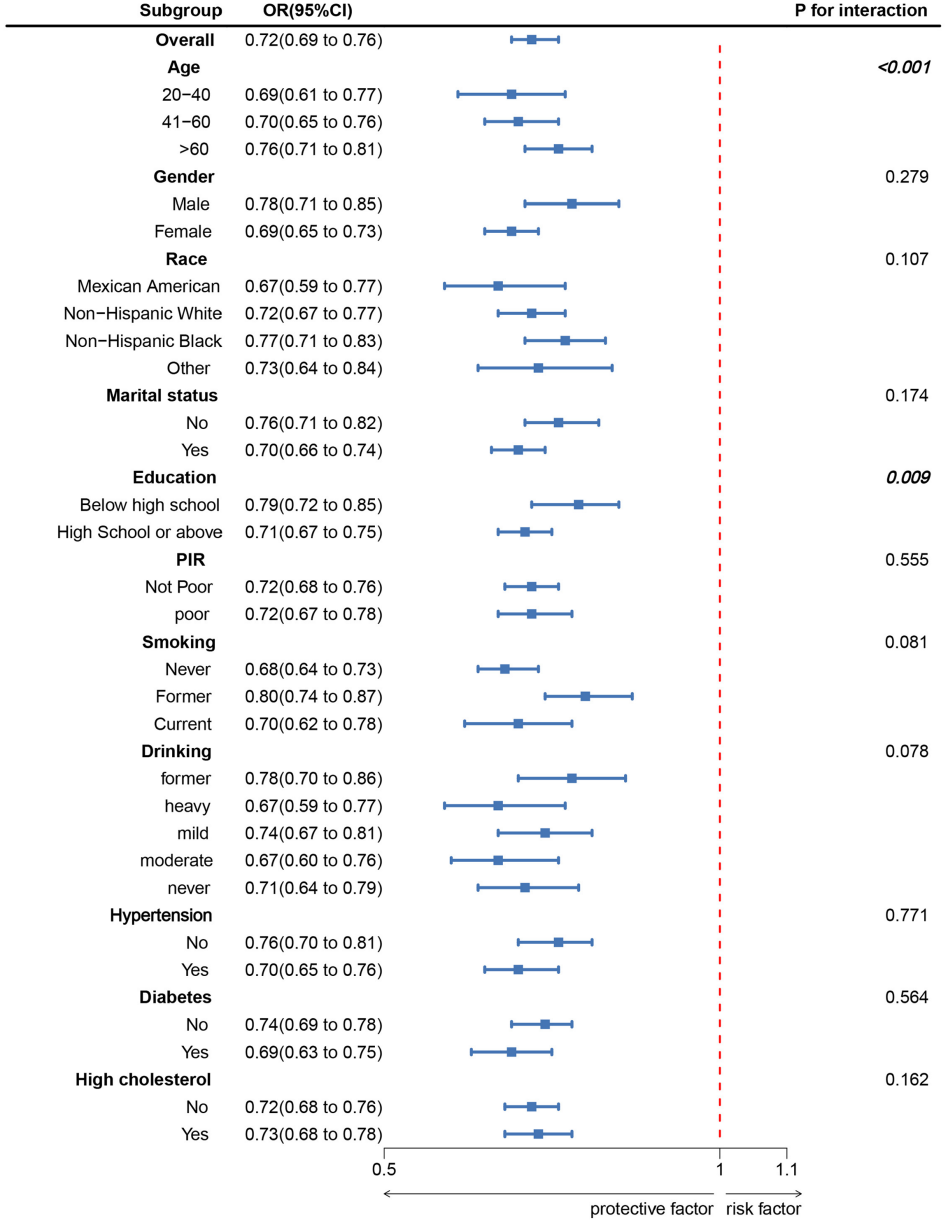
Subgroup analysis between LC9 and OAB. ORs were calculated as per 10-unit increase in LC9. Analyses were adjusted for age, gender, education level, marital, PIR, race, smoking, drinking, hypertension, diabetes, and high cholesterol.

Subgroup analyses were conducted based on age, sex, race, marital status, education level, poverty-to-income ratio (PIR), smoking, alcohol consumption, hypertension, diabetes, and hypercholesterolemia (Figure 3). The results indicated a significant negative correlation between LC9 scores and OAB prevalence across all subgroups. Furthermore, a significant interaction was observed between LC9 and both age and education level (*p* < 0.05).

### Mediation effect

**Figure 4:**
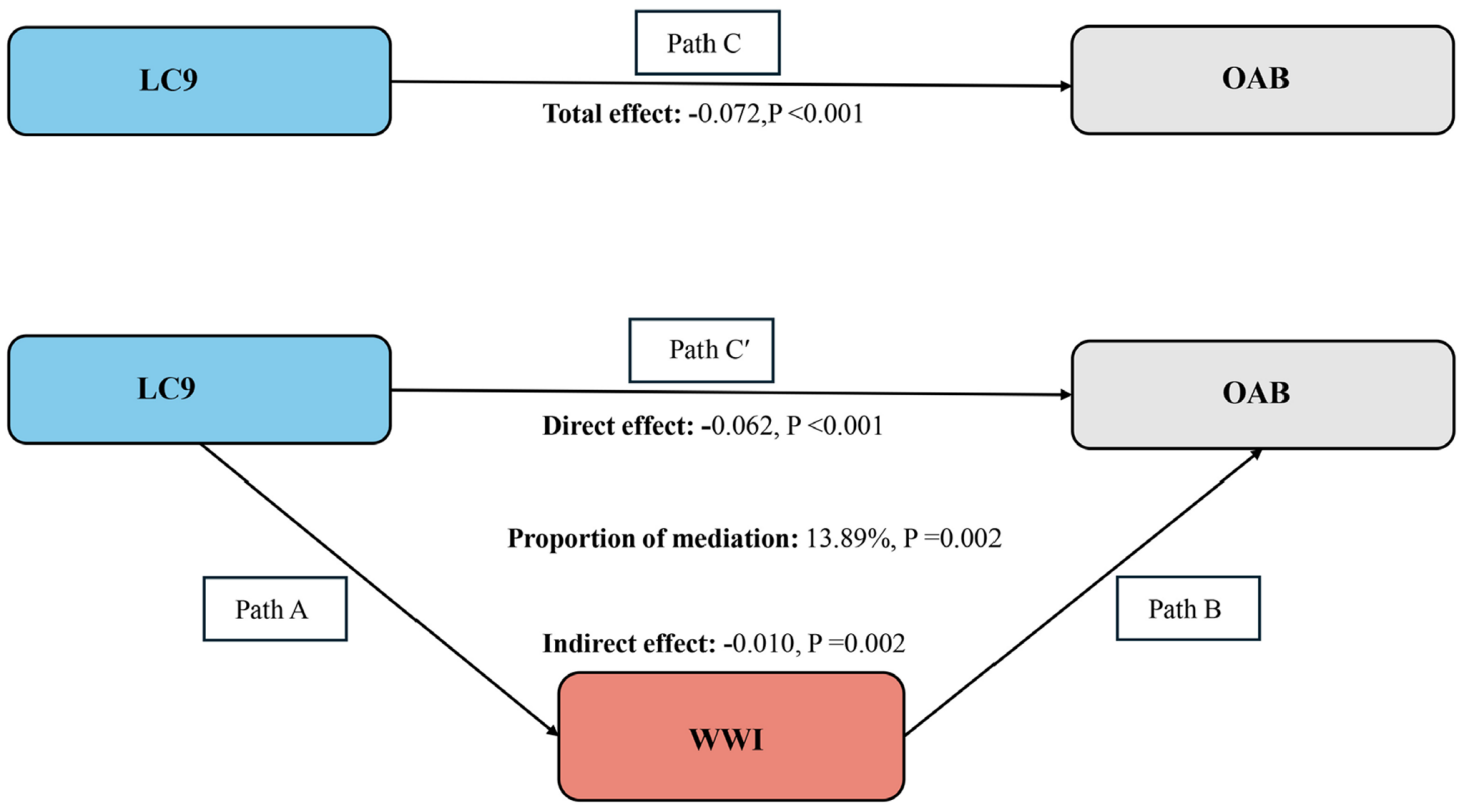
Schematic diagram of the mediation effect analysis. Path C indicates the total effect; path C′ indicates the direct effect. The indirect effect is estimated as the multiplication of paths A and B (path A*B). The mediated proportion is calculated as indirect effect/ (indirect effect + direct effect) × 100%. Abbreviation: LC9, Life’s Crucial 9; OAB, overactive bladder; WWI, weight-adjusted-waist index. Analyses were adjusted for age, gender, education level, marital, PIR, race, smoking, drinking, hypertension, diabetes, and high cholesterol.

The mediation model is illustrated in Figure 4, with LC9, OAB, and WWI serving as the independent variable, dependent variable, and mediator variable, respectively. As shown in Table 3, a significant correlation was observed between LC9 and WWI after adjusting for other covariates (*β* = −0.25, 95% CI: −0.26, −0.24). Following the adjustment for all covariates, the mediating effect of WWI was evident (indirect effect = −0.010, *p* = 0.002; direct effect = −0.062, *p* < 0.001), resulting in a mediation proportion of 13.89% (*p* = 0.002). Therefore, WWI can be regarded as a mediating factor in the association between LC9 and OAB.

**Table 3:** Multivariate linear regression of LC9 and WWI.

	*β*	95%CI	*p*-value
LC9-WWI	−0.25	(−0.26, −0.24)	<0.001

Adjusted for age, gender, education level, marital, PIR, race, smoking, drinking, hypertension, diabetes, and high cholesterol.

## Discussion

In this study, we investigated 25,319 participants from NHANES 2005–2018, demonstrating a negative correlation between LC9 and the prevalence of OAB, while a positive correlation was observed between WWI and OAB. Furthermore, the mediation analysis indicated that WWI partially mediated the association between LC9 and OAB. Notably, a significant interaction was found between LC9 scores and both age and education level.

To our knowledge, this is the first study to investigate the association between LC9 and OAB mediated by WWI. Previous research has indicated a negative correlation between cardiovascular health (CVH), quantified by the LE8 score, and the prevalence of OAB. Additionally, WWI has been shown to predict the incidence of OAB, with obesity management contributing to a reduced risk of OAB (28, 29), findings that align with our study. However, these studies did not consider the impact of mental health on cardiovascular health and OAB. Our research not only confirms this association but also further explores the relationship between Life’s Essential 9 and OAB, emphasizing the significance of mental health in cardiovascular health. Mental health encompasses various aspects, such as depression, anxiety, and chronic, and traumatic stress, all of which are related to cardiovascular disease risk.

The components of LC9 influence OAB through several mechanisms. The dietary component of LC9, particularly weight control, increased fiber intake, and adequate hydration, may indirectly affect OAB by reducing inflammation, improving metabolic health, and promoting bladder health (15). An increase in dietary fiber and antioxidants helps reduce bladder inflammation and alleviate OAB symptoms. Diets rich in fruits, vegetables, and whole grains can lower chronic low-grade inflammation, which may contribute to the alleviation of OAB symptoms. Physical activity impacts OAB through various pathways. Increased physical activity can reduce OAB symptoms by enhancing core muscle strength, improving pelvic muscle tone, and restoring normal bladder function. Additionally, regular exercise improves blood circulation, reduces cardiovascular disease risk, and decreases the stress and discomfort associated with OAB (30). Physical activity also helps control body weight, improving fat distribution and reducing abdominal fat accumulation, thereby relieving pressure on the bladder. Sleep quality plays a crucial role in OAB. Insufficient or poor-quality sleep leads to increased activation of the nervous system, exacerbating symptoms such as frequent urination and urgency. The sleep component of LC9 emphasizes good sleep habits and adequate sleep duration, which helps regulate the sympathetic nervous system and reduce bladder overactivity. Moreover, quality sleep promotes restorative rest and alleviates anxiety or depression induced by OAB, thereby easing symptoms.

Interaction analysis suggests that LC9 may interact with age and education level in influencing OAB. The CVH score appears to have a greater impact on individuals with education beyond high school. This might be because they are more likely to recognize OAB as a medical condition and seek solutions compared to those with lower educational attainment. Cultural barriers, such as stigma and health beliefs, might also play a role (31, 32). Aging alone is considered a major risk factor for OAB, leading to anatomical and functional changes such as reduced bladder capacity and compliance. Additionally, with age, the detrusor muscle of the bladder shows decreased expression of muscarinic receptors and neural innervation, resulting in reduced cholinergic transmission (33). Imaging studies further reveal age-related differences in supraspinal control of non-neurogenic overactive bladder (OAB). In younger patients with OAB, areas like the insula and anterior cingulate cortex (ACC) show moderate responses at low bladder volumes but increase at higher volumes, while the opposite is true for older patients (34).

Lower urinary tract symptoms (LUTS) are closely associated with cardiovascular disease (CVD), which has been proposed as a potential risk factor for the progression and severity of LUTS (35). The mechanisms linking these conditions are multifaceted and complex. The pathogenesis of overactive bladder (OAB) is closely related to various cardiovascular risk factors, with oxidative stress (OS) being a fundamental mechanism of CVD. Worsening oxidative stress can alter the function and/or structure of the bladder, urethra, and prostate, leading to LUTS (36). Since the introduction of metabolic syndrome (MetS) in 1977, it has emerged as a significant precursor to CVD and other chronic diseases, with obesity and insulin resistance (IR) considered its core components (37, 38). Recent studies have shown an association between OAB and MetS in women (16, 39). The overactivity of the sympathetic nervous system, pro-inflammatory states, OS, and other pathological conditions related to MetS may also be linked to OAB (40). Regarding IR, it is associated with sympathetic overactivity, a key neuropathological finding in idiopathic OAB patients (41). Furthermore, an animal study has demonstrated that hyperinsulinemia resulting from IR can induce bladder smooth muscle relaxation, dependent on the activation of the PI3K/AKT/eNOS signaling pathway within the mucosal layer, subsequently releasing NO to relax detrusor smooth muscle (42).

The occurrence of lower urinary tract symptoms (LUTS) is multifactorial, with obesity being one of the contributing factors (43). A randomized controlled trial involving 77 OAB patients demonstrated that reducing abdominal fat alleviated OAB symptoms, a finding supported by another study (13, 44). While earlier studies used body mass index (BMI) as a measure of disease, recent research has shifted toward identifying physiological and metabolic dysfunction markers of adipose tissue, with a greater focus on regional fat, such as visceral fat (45). The waist-to-weight ratio (WWI) better reflects visceral fat than BMI. Accumulation of visceral adipose tissue (VAT) leads to chronic inflammation and insulin resistance. VAT infiltrated by inflammatory macrophages becomes a source of low-grade chronic inflammation, which is associated with the OAB syndrome. One study showed that inflammation markers in OAB patients were significantly higher than in controls (46, 47). Previous research suggested that poorer cardiovascular health (CVH), quantified by the Life’s Simple 7 (LS7) score, is associated with increased visceral fat area and elevated HOMA-IR levels (48). LC9, as a new indicator for assessing CVH, emphasizes mental health, with anxiety and depression being linked to LUTS. When anxiety and depression occur together, they seem to have an additive effect on the association with LUTS. Emotional disorders may result in LUTS, and conversely, LUTS may make patients more susceptible to emotional disorders (49).

The potential mechanisms through which WWI mediates the relationship between LC9 and OAB are as follows: An increase in WWI may indicate a rise in visceral fat accumulation, which is associated with metabolic disturbances such as insulin resistance, hyperglycemia, and dyslipidemia (50). These metabolic factors could contribute to bladder dysfunction by altering the balance of neurotransmitters (e.g., acetylcholine, norepinephrine) involved in bladder contraction, leading to OAB. Furthermore, visceral fat has been shown to secrete pro-inflammatory cytokines (e.g., TNF-*α*, IL-6), which can activate inflammatory pathways in bladder smooth muscle cells, increasing bladder sensitivity and contributing to OAB. Additionally, improvements in the health behaviors recommended by LC9, such as increased physical activity and dietary changes, may help reduce abdominal fat (51). This, in turn, could indirectly reduce WWI and improve metabolic health, thereby lowering the risk of OAB.

This study has several strengths: (1) It is the first to examine the correlation between LC9 and the prevalence of OAB in an American population, indicating significant potential for LC9 as a diagnostic and assessment tool for OAB. It provides new recommendations for OAB diagnosis and evaluation. (2) WWI is introduced as a new indicator for assessing visceral fat content, outperforming traditional anthropometric methods in screening for OAB, thus facilitating low-cost identification of OAB in clinical settings. (3) The study includes a large sample size, utilizing population data from NHANES 2005–2018, which allows for a nationally representative characterization of the population. (4) By constructing various models and conducting subgroup analyses to adjust for confounding factors, the study demonstrates a strong positive correlation between LC9 and OAB. The results are robust and reliable. (5) Subgroup analysis reveals that age and education level may be potential modifiers of the relationship between LC9 and OAB (*p* < 0.05), suggesting that these factors influence the association between CVH and OAB prevalence. This highlights the need for further research to validate these associations.

Despite the significance of this study, several limitations need to be acknowledged: (1) This is a cross-sectional study, which cannot establish a causal relationship between LC9 and the prevalence of OAB. Further large-scale, prospective studies are needed to clarify the causality between LC9 and OAB. (2) NHANES employs a complex, multistage, stratified probability sampling method, theoretically representing the U.S. noninstitutionalized population. However, certain populations, such as hospitalized patients and residents of long-term care facilities, were excluded, potentially limiting the generalizability of our findings to the entire U.S. population. Moreover, selection bias may arise from individuals who did not participate in the survey or complete specific assessments. (3) The diagnosis of OAB in this study relies on the OABSS score derived from nocturia and urgency urinary incontinence symptoms recorded by NHANES. The frequency of these symptoms may be subject to recall bias. (4) This study utilized NHANES data, which, while providing a large sample size, may be subject to potential confounding effects and issues related to multiple dependent variables. Given that each LC9 component (such as diet, physical activity, and sleep) is measured at different levels, there may be interrelationships between these variables that were not fully accounted for. As a result, this study may not have fully isolated the independent effects of each component on OAB outcomes. Additionally, due to variations in data quality, the selection of appropriate statistical methods is crucial to ensure the accuracy of the results, particularly in large sample datasets where confounding factors may influence the final analysis. Future studies could consider further exploring the potential interactions among these variables and applying more refined statistical methods to mitigate the impact of confounding effects. (5) Additionally, one of the limitations of this study is the use of the OABSS cutoff value of 3 to define overactive bladder (OAB). While this cutoff is widely used in existing literature and has been validated in several studies, we acknowledge that it has certain limitations, especially in its applicability across different populations and clinical contexts. Specifically, the OABSS cutoff value of 3 classifies OAB based on symptom frequency and severity, but this simplified threshold may not fully capture the complexity and variability of OAB symptoms, particularly in individuals with mild or intermittent symptoms. For example, increased nocturia or occasional episodes of urgency/urge incontinence may be indicative of OAB, but these symptoms might not reach the OABSS cutoff of 3, potentially leading to underdiagnosis of OAB in some patients. Furthermore, the OABSS cutoff of 3, being a quantitative measure, does not account for individual differences in symptom perception and the subjective impact on quality of life. Different patients may perceive and report symptoms differently, and a single cutoff value may not fully reflect the impact of symptoms on an individual’s daily life. As such, our study may either underestimate or overestimate the true prevalence of OAB. Additionally, since the OABSS was not originally designed to capture all clinical aspects of OAB, relying solely on this scoring system may overlook other factors influencing OAB symptoms, such as urodynamic abnormalities or changes in bladder capacity. Therefore, future research should consider combining the OABSS with other clinical assessment tools or symptom scales to provide a more comprehensive evaluation of the symptom burden and quality of life in OAB patients. Given these limitations, we suggest that future studies explore whether more refined diagnostic criteria are needed, considering not only the frequency and severity of symptoms but also their duration and impact on quality of life. Longitudinal data and more precise diagnostic standards will help further validate the definition and diagnostic methods for OAB.

## Conclusion

In conclusion, we identified a significant negative correlation between LC9 and OAB, with WWI as a partial mediator. This finding underscores the potential link between cardiovascular health and OAB, highlighting the importance of obesity management in this context. Our study provides new insights into the prevention and management of OAB, emphasizing that a comprehensive approach to improving cardiovascular health and addressing obesity may help reduce the prevalence of OAB. Therefore, prospective studies are necessary to provide more definitive evidence regarding the underlying mechanisms. Additionally, future research could further explore other potential risk factors, such as mental health disorders, which may also influence this relationship.

## Data Availability

The datasets presented in this study can be found in online repositories. The names of the repository/repositories and accession number(s) can be found at: https://www.cdc.gov/nchs/nhanes/index.htm.

## Footnotes

^1^ https://www.cdc.gov/nchs/nhanes/index.htm
